# Adrenal tumours in patients with pathogenic *APC* mutations: a retrospective study

**DOI:** 10.1186/s13053-024-00289-1

**Published:** 2024-09-03

**Authors:** Lyman Lin, Victoria Beshay, Finlay Macrae

**Affiliations:** 1 Department of Colorectal Medicine and Genetics, Royal Melbourne Hospital, Parkville, VIC Australia; 2 Department of Molecular Diagnostic Pathology, Peter MacCallum Cancer Centre, Melbourne, VIC Australia; 3 Department of Medicine, University of Melbourne, Parkville, VIC Australia

**Keywords:** Genetics, Colorectal cancer, *APC*, Familial adenomatous polyposis, Adrenal tumour

## Abstract

**Background:**

Adrenal tumours are associated with familial adenomatous polyposis (FAP). In the literature, most studies use the clinical definition of FAP (more than 100 adenomatous polyps found in endoscopic studies). However, not all patients that meet clinical criteria for FAP carry pathogenic mutations in the adenomatous polyposis coli (*APC*) gene, as there is genetic heterogeneity responsible for FAP with the polyposis sometimes explained by genetic and environmental factors other than pathogenic *APC* mutations. Reciprocally, not all the patients with pathogenic *APC* variants will fulfil the classic criteria of FAP.

**Objective:**

This study aims to investigate the characteristics of adrenal tumours in patients with pathogenic or likely pathogenic *APC* variants and explore the hormonal function of these patients.

**Method:**

This is a retrospective cohort study. Patients with pathogenic or likely pathogenic *APC* variants were recruited and their radiological assessments were reviewed. Patient demographic data, *APC* variants, adrenal mass characteristics and hormonal testing results were collected.

**Result:**

The prevalence of adrenal mass was 26.7% (24/90) among patients with pathogenic or likely pathogenic *APC* variants. Using the classic definition, the prevalence was 32.4% (22/68). Four patients had adrenal hormone testing, two of which had Conn’s syndrome and two had nonspecific subclinical results.

**Conclusion:**

In our cohort, the prevalence of adrenal tumours among patients with pathogenic and likely pathogenic APC mutations is at least twice to three times higher than the general population prevalence reported from international population-based studies. The hormonal functions of patients with pathogenic *APC* variants and adrenal tumours can be investigated with routine testing in further research.

## Background

Familial adenomatous polyposis (FAP) is an autosomal dominant genetic disorder that is caused by a germline mutation in the adenomatous polyposis coli (*APC*) gene [1, 2]. The classic clinical sign is the development of more than 100 colorectal adenomas throughout the colon with or without extraintestinal manifestations [2]. The incidence of FAP at birth is 1 in 8,300, based on a genetic study in the United Kingdom [3].

One of the recognised extraintestinal manifestations of FAP is adrenal tumours. Here we use the term “tumour” as a mass lesion identified on imaging or through surgical pathology. Most are considered to be adenomas [4]. The prevalence of adrenal tumours among FAP patients has been estimated to be 16% from a Canadian study [4], in comparison to a 7% prevalence among the public in Canada [5]. This result is consistent with small case series in the US, UK and Netherlands [6–8].

There are two main clinical concerns regarding adrenal tumours: the malignancy potential, and hormonal function abnormalities. From the four largest retrospective studies so far, the malignancy rate of adrenal tumours found in FAP patients seems to be consistent with those in the general population [4, 6–8]. Therefore, the authors from the four registries suggest that no special considerations are required for the surveillance and management of adrenal tumours for FAP patients. Regarding the functional status of these lesions, hyperfunction appears to be uncommon, based on the small group of patients who have undergone adrenal hormone testing [4, 8].

In the literature, most of the studies use the clinical definition of FAP, where more than 100 colonic polyps were found in endoscopic studies. However, not all patients meeting clinical criteria for FAP carry pathogenic *APC* mutations, as there is genetic heterogeneity responsible for FAP with the polyposis explained by genetic and environmental factors other than pathogenic *APC* mutations. Reciprocally, not all the patients with pathogenic *APC* variants will fulfil the classic criteria of FAP. FAP is increasingly now a genotypically defined disorder, as variability of the phenotype even in those with pathogenic *APC* mutations is common.

This study aims to investigate the characteristics of adrenal tumours in patients with pathogenic or likely pathogenic *APC* variants and the degree of adrenal hormonal testing among these patients.

## Method

Records of patients aged 18 to 90, who attended the Parkville Familial Cancer Clinic and who carried a constitutional pathogenic or likely pathogenic *APC* variant were reviewed.

The variant of *APC* was detected using the Colorectal and Polyps assay by the Peter MacCallum Cancer Institute DNA diagnostic laboratory that targeted familial cancer-related genes including *APC*. Targeted gene sequencing of coding regions and splice sites was performed on DNA extracted from blood. Libraries were prepared and enriched using SureSelect XT target enrichment. Indexed libraries were pooled and sequenced to a targeted coverage of 700 reads/base (Illumina Next Seq500, 2*75 bp). Sqlinerv0.5 was used to generate aligned reads and call variants against the hg19 human reference genome. The sequence variants were classified according to ClinVar or InSiGHT LOVD database.

Patients with benign variants, likely benign variants, variants of unknown significance or where no *APC* variants were identified were excluded. Patients without available radiological reports in our database were also excluded. Data were collected regarding patients’ demographics, age of diagnosis, *APC* genotypes, presence and characteristics of adrenal tumours, CT or MRI findings and findings from adrenal hormone testing. Patients with adrenal tumours were defined as those who had radiological evidence of an adrenal lesion equal to or larger than 1 cm in maximal diameters. This is in concordance with previous studies [5–9].

The endocrinological investigations of interest included but were not limited to morning serum cortisol level, 24-hour urine cortisol level, dexamethasone suppression test, serum/urine metanephrine level, aldosterone to renin ratio and gonadocorticoids level.

Descriptive statistics were computed for all variables using medians and percentiles for continuous factors and frequencies and percentages for categorical variables. A Chi-sqaure test was performed to investigate whether there was an association between genotype and presence of adrenal tumours. The analyses were performed by using IBM SPSS software, version 25.

## Result

**Fig. 1:**
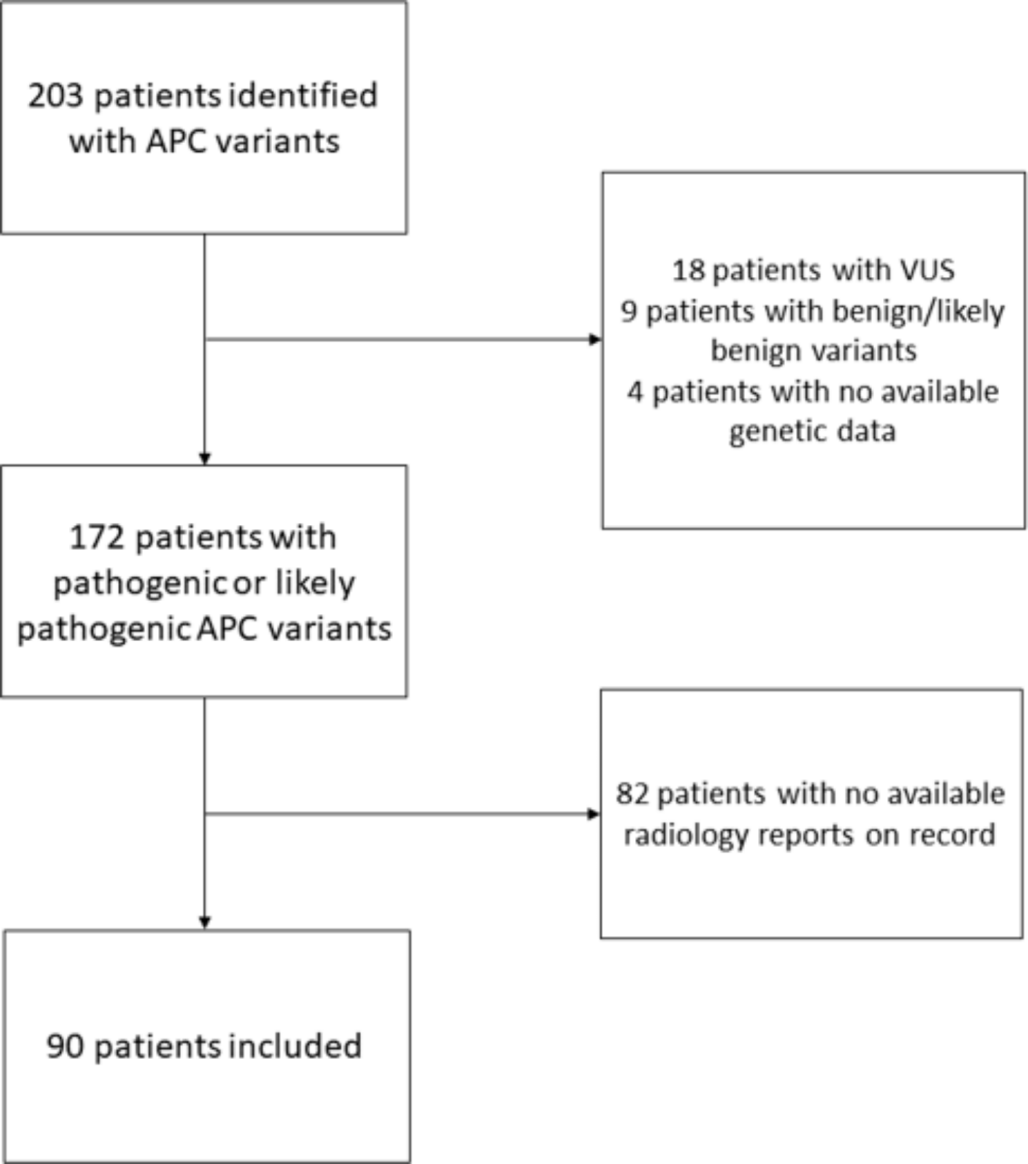
Flow chart of the patient selection process

172 patients had pathogenic or likely pathogenic *APC* variants. 82 of the 172 patients were externally managed and thus no radiology records are available in our database. In total, 90 patients met the inclusion criteria with pathogenic or likely pathogenic variants and available CT/MRI scans covering the adrenal glands’ regions (see Fig. 1). The CT and MRI scans were conducted from January 2009 to January 2023. The majority of CT or MRI scans were conducted for colorectal cancer staging, desmoids tumour screening or planning for colorectal surgery.

66 out of 90 (73.3%) had normal adrenal glands and 24 (26.7%) had adrenal tumours. The demographic information of these patients can be seen in Table  1. In total, 34 adrenal tumours were found in the 24 patients. The characteristics of these tumours are summarised in Table 2. No malignant features of adrenal glands were detected among the cohort based on CT or MRI scans. No significant correlation is found between genotype and the presence of adrenal tumours (*p* = 0.552).

The classic definition of FAP was the development of more than 100 colorectal adenomas throughout the colon detected by endoscopic studies [2]. Notably, 4 patients in our cohort that fulfil the clinical diagnosis of classic FAP had non-pathogenic *APC* variants (one with likely benign variants and three with VUS), and they were excluded as per our criteria. None of them have radiological evidence of adrenal masses on scans. In contrast, 24 patients with pathogenic or likely pathogenic *APC* variants did not strictly fulfil the classic definition of FAP but were considered in our study. 13 of them had available radiological records and 2 out of 13 (15.4%) had evidence of adrenal tumours. When the classic definition of FAP is used 22 out of 68 (32.4%) FAP patients had adrenal tumours and 46 (67.6%) had normal adrenal glands.

Routine hormonal screening was not conducted for adrenal incidentaloma in our practice. There were only four patients who had adrenal hormone testing for various reasons (Table 3). Two patients were tested as part of a secondary hypertension screen and both had Conn’s Syndrome. The other two were tested because they presented with systemic symptoms of weight loss and fatigue in the context of their adrenal tumours. They both had mild abnormalities in hormone testing, not considered responsible for their clinical presentation.

**Table 1:** Characteristics of adrenal tumours among patients with pathogenic or likely pathogenic mutations

PARAMETERS	PATIENTS, *n* = 90	
	No adrenal tumours, *n* = 66	Adrenal tumours, *n* = 24
Sex		
Male	36 (54.5%)	11 (45.8%)
Female	30 (45.5%)	13 (54.2%)
The median age of FAP diagnosis, years (IQR)	22 (18–31)	29 (20–43)
The median age when the radiological assessment was conducted, years (IQR)	39 (29–50)	49 (35–61.75)
Modality		
CT	49 (74.2%)	17 (70.8%)
MRI	17 (25.8%)	7 (29.2%)
Laterality		
Left only		11 (45.8%)
Right only		3 (12.5%)
Bilateral		9 (37.5%)
Unknown		1 (4.2%)
Median maximal diameter, mm (IQR)		17 (12.5–23)
Type of adrenal tumours		34 tumours in total
Adenoma		25 (73.5%)
Myelolipoma		3 (8.8%)
Indeterminate		6 (17.6%)

**Table 2:** Clinical details of the cases

Sex	Age	Variant	Variant type	Pathogenicity	Adrenal mass type	Maximal diameter (mm)
Male	47	NM_000038.5:c.1958 + 1_1958 + 2dupGT	Splice donor variant	Likely Pathogenic	Adrenal adenoma	20
Male	34	NM_000038.5:c.531 + 1G > T	Splice donor variant	Pathogenic	Bilateral adrenal adenomas	L) 17, R) 12
Male	52	NM_000038.5:c.3924dup	Frameshift	Pathogenic	Bilateral adrenal adenomas	L) 26, R) 33
Male	29	NM_000038.5:c.5826_5829del	Frameshift	Pathogenic	Bilateral adrenal adenomas	R) 22, L) unknown
Female	36	NM_000038.5:c.3444_3447del	Frameshift	Pathogenic	Bilateral adrenal adenomas	L) 16, R) 32
Female	62	NM_000038.5:c.1885_1886insA	Frameshift	Pathogenic	Bilateral adrenal adenomas (3 in total)	L) inferior 31, L) superior 12, R) 6
Male	24	NM_000038.5:c.2805 C > A	Nonsense	Pathogenic	Left adrenal adenoma	20
Female	30	NM_000038.5:c.5952_5955del	Frameshift	Pathogenic	Left adrenal adenoma	12
Female	14	NM_000038.5:c.487 C > T	Nonsense	Pathogenic	Bilateral indeterminate adrenal masses	L) 15, R) 10
Female	29	NM_000038.5:c.1259_1269del	Frameshift	Pathogenic	Left adrenal adenoma	18
Male	20	NM_000038.5:c.904 C > T	Nonsense	Pathogenic	Bilateral adrenal adenomas	L) 12, R) 12
Female	63	NM_000038.5:c.7016_7064del	Frameshift	Pathogenic	Left adrenal adenoma	15
Female	19	NM_000038.5:c.2805 C > A	Nonsense	Pathogenic	Left adrenal myelolipoma	17
Female	43	NM_000038.5:c.3183_3187del	Frameshift	Pathogenic	Left adrenal myelolipoma and right adrenal adenoma	L) unknown, R) 15
Female	40	**		Pathogenic	Left adrenal nodule	
Female	26	NM_000038.5:c.423-1G > A	Splice donor variant	Pathogenic	Left adrenal myelolipoma	41
Female	46	NM_000038.5:c.3183_3187del	Frameshift	Pathogenic	Left adrenal nodule	13
Male	20	NM_000038.5:c.487 C > T	Nonsense	Pathogenic	Left adrenal nodule	35
Female	N/A	NM_000038.5:c.487 C > T	Nonsense	Pathogenic	Right adrenal adenoma	21
Female	17	NM_000038.5:c.348_352del	Frameshift	Pathogenic	Right adrenal adenoma	11
Male	43	NM_000038.5:c.348_352del	Frameshift	Pathogenic	Right adrenal nodule	15
Male	18	Truncated protein from exon 15*		Pathogenic	Bilateral adrenal adenomas	L) unknown, R) 18
Male	23	NM_000038.5:c.2805 C > A	Nonsense	Pathogenic	Left adrenal adenoma	12
Male	18	NM_000038.5:c.2805 C > A	Nonsense	Pathogenic	Left adrenal adenoma	29

*Patient had protein testing only when genetic sequencing was not widely available at the time in clinical practice

**Patients had the genetic testing externally. The exact variant was not available but was labelled as pathogenic on the record

**Table 3:** Adrenal hormone testing results

Age	Type of adrenal mass	Early morning cortisol level, nmol/L (Ref: 100–540 nmol/L)	Urine metanephrine/creatinine ratio (Ref: <0.10 mmol/mol)	Plasma metanephreines, pmol/L (Ref: < 500pmol/L)	Plasma normetanephrine, pmol/L (Ref: <900 pmol/L)	Aldosterone/renin ratio (Ref: <70)	17-hydroxyprogesterone, nmol/L (Ref: <70)	Adrenocorticotropic hormone, ng/L (Ref: 7.2–63.3 ng/L)	Estradiol, pmol/L	Follicle-stimulating hormone, IU/L	Luteinising hormone, IU/L	Indication for testing
75	Bilateral adrenal adenomas	67	-	362	1148	42	0.4	22.1	< 18	2.4	0.5	Fatigue in the context of adrenal tumour
46*	Bilateral adrenal adenomas	153	0.14	148	674	96	-	-	-	-	-	Secondary hypertension screen
36	Left adrenal adenomas	78	-	-	-	45	-	-	142	13	4	Fatigue in the context of adrenal tumour
44*	Bilateral adrenal adenomas	-	-	-	-	98	-	-	-	-	-	Secondary hypertension screen

*: both patients were diagnosed with Conn’s syndrome

## Discussion

The inclusion criteria for patients in this study are based on the pathogenicity of the *APC* variants they carry, in contrast to other international studies where a clinical definition of FAP was used (> 100 adenomatous polyps in endoscopic studies). In our cohort, some patients presented with polyposis on colonoscopy but had VUS or benign *APC* variants. These patients were excluded from the study to limit the heterogeneity of the studied population. Patients with pathogenic *APC* variants who did not strictly fulfil the classic clinical criteria of FAP in endoscopic studies were included as per our criteria. Rather than regarding adrenal tumours as an extraintestinal manifestation of FAP, we focus on whether the pathogenicity of *APC* variants would affect the formation of adrenal tumours.

There is no Australian data on the prevalence of adrenal tumours in FAP. From recent international studies, the prevalence of adrenal tumours is 3–10% in the general population [10–14] and 7.4–26% among FAP patients defined phenotypically [4, 6–8]. The prevalence of adrenal tumours among patients with pathogenic or likely pathogenic *APC* mutations in this study (26.7%) is so far the highest.

There was no significant correlation between genotype and the presence of adrenal tumours among those with pathogenic variants. This result is consistent with the Canadian study [4]. Most of the variants in our patients with adrenal tumours involve frameshift or premature stop codons, typical of pathogenic variants described in *APC* in the literature and on the LOVD and ClinVar databases.

No malignant features of adrenal glands were detected among the cohort based on CT or MRI scans. This is consistent with the four previous retrospective studies where only two cases of malignancy were found among them, and it was hypothesised that the prevalence of malignant adrenal tumours among FAP is similar to that in the general population [4, 6–8].

There are some limitations identified in this study. This study is retrospective in nature and a control group was not included. Also, the prevalence of adrenal tumours in the general Australian population is unavailable. Therefore, we were unable to directly compare the prevalence of adrenal tumours in our cohort against the Australian population.

The CT or MRI scans were conducted at no specific patient age. Rather, they were done for various reasons, including screening for desmoid tumours, staging for malignancy, and investigating abdominal pain. The percentage of the patients who received the scans due to presentation with gastrointestinal symptoms was similar between those with and without adrenal tumours (41.7% vs. 43.9%) supporting the likelihood that the findings from this study were representative of all patients with FAP. No initial CT or MRI scans of the patients in our cohort were conducted related to clinical suspicion of adrenal tumours (like symptoms of hormonal disturbance). All adrenal lesions we found were considered to be adrenal incidentalomas.

No routine adrenal hormonal testing was conducted for adrenal incidentalomas. Only four patients in this study had adrenal hormonal testing due to secondary hypertension screening and constitutional symptoms of weight loss and fatigue in the context of the adrenal tumours. Thus, it is difficult to assess the pattern of hormonal function for patients with pathogenic *APC* variants and adrenal tumours. In the general population, isolated or combined subclinical hormonal abnormalities can occur in 37.4% of patients with adrenal tumours [15]. However, no studies in the literature systematically investigate hormonal abnormalities among FAP patients with adrenal tumours. Given the increased prevalence of adrenal tumours among those with pathogenic APC variants, future research can investigate the functional consequences of these adrenal tumours. This will provide meaningful information on whether routine hormonal testing on this cohort will be beneficial.

## Conclusion

The prevalence of adrenal tumours among patients with pathogenic and likely pathogenic *APC* mutations in our cohort is likely to be twice to three times higher than the general population prevalence reported from international population-based studies. More data on adrenal tumours in classic FAP patients with non-pathogenic *APC* variants is needed, like in those with *MUTYH* and *HTHL1* variants. The hormonal functions of patients with pathogenic *APC* variants and adrenal tumours should be investigated in further research to better understand whether there are any clinical endocrine implications of the increased prevalence of adrenal tumours in these patients.

## Data Availability

No datasets were generated or analysed during the current study.

## References

[1] Dinarvand P, Davaro EP, Doan JV, Ising ME, Evans NR, Phillips NJ, Lai J, Guzman MA. Familial adenomatous polyposis syndrome: an update and review of Extraintestinal manifestations. Arch Pathol Lab Med. 2019;143(11):1382–98. 31070935 10.5858/arpa.2018-0570-RA

[2] Aihara H, Kumar N, Thompson CC. Diagnosis, surveillance, and treatment strategies for familial adenomatous polyposis: rationale and update. Eur J Gastroenterol Hepatol. 2014;26(3):255–62. 24161962 10.1097/MEG.0000000000000010 PMC5019104

[3] Reed TE, Neel JV. A genetic study of multiple polyposis of the colon with an appendix deriving a method of estimating relative fitness. Am J Hum Genet. 1955;7(3):236–63. 13258558 PMC1716610

[4] Shiroky JS, Lerner-Ellis JP, Govindarajan A, Urbach DR, Devon KM. Characteristics of adrenal masses in familial adenomatous polyposis. Dis Colon Rectum. 2018;61(6):679–85. 29377868 10.1097/DCR.0000000000001008

[5] Granger P, Genest J. Autopsy study of adrenals in unselected normotensive and hypertensive patients. Can Med Assoc J. 1970;103(1):34–6. 5424294 PMC1930349

[6] Marchesa P, Fazio VW, Church JM, McGannon E. Adrenal masses in patients with familial adenomatous polyposis. Dis Colon Rectum. 1997;40(9):1023–8. 9293929 10.1007/BF02050923

[7] Smith TG, Clark SK, Katz DE, Reznek RH, Phillips RK. Adrenal masses are associated with familial adenomatous polyposis. Dis Colon Rectum. 2000;43(12):1739–42. 11156460 10.1007/BF02236860

[8] Kallenberg FGJ, Bastiaansen BAJ, Nio CY, Soeters MR, Boermeester MA, Aalfs CM, Bossuyt PMM, Dekker E. Adrenal lesions in patients with (attenuated) familial adenomatous polyposis and MUTYH-Associated polyposis. Dis Colon Rectum. 2017;60(10):1057–64. 28891849 10.1097/DCR.0000000000000809

[9] Chandrasekar T, Goldberg H, Klaassen Z, Wallis CJD, Woon DTS, Herrera-Caceres JO, Kulkarni GS, Fleshner NE. The who, when, and why of primary adrenal malignancies: insights into the epidemiology of a rare clinical entity. Cancer. 2019;125(7):1050–9. 30561782 10.1002/cncr.31916

[10] Young WF Jr. Clinical practice. The incidentally discovered adrenal mass. N Engl J Med. 2007;356(6):601–10. 17287480 10.1056/NEJMcp065470

[11] Iñiguez-Ariza NM, Kohlenberg JD, Delivanis DA, Hartman RP, Dean DS, Thomas MA, Shah MZ, Herndon J, McKenzie TJ, Arlt W, Young WF Jr, Bancos I. Clinical, biochemical, and radiological characteristics of a single-Center Retrospective Cohort of 705 large adrenal tumors. Mayo Clin Proc Innov Qual Outcomes. 2017;2(1):30–9. 30225430 10.1016/j.mayocpiqo.2017.11.002 PMC6124341

[12] Mao JJ, Dages KN, Suresh M, Bancos I. Presentation, disease progression and outcomes of adrenal gland metastases. Clin Endocrinol (Oxf). 2020;93(5):546–54. 32569405 10.1111/cen.14268 PMC7875181

[13] Tallis PH, Rushworth RL, Torpy DJ, Falhammar H. Adrenal insufficiency due to bilateral adrenal metastases - a systematic review and meta-analysis. Heliyon. 2019;5(5):e01783. 31193734 10.1016/j.heliyon.2019.e01783 PMC6541881

[14] Lubomski A, Falhammar H, Torpy DJ, Rushworth RL. The epidemiology of primary and secondary adrenal malignancies and associated adrenal insufficiency in hospitalised patients: an analysis of hospital admission data, NSW, Australia. BMC Endocr Disord. 2021;21(1):141. 34217233 10.1186/s12902-021-00787-6 PMC8254950

[15] Bernini GP, Moretti A, Oriandini C, Bardini M, Taurino C, Salvetti A. Long-term morphological and hormonal follow-up in a single unit on 115 patients with adrenal incidentalomas. Br J Cancer. 2005;92(6):1104–9. 15770213 10.1038/sj.bjc.6602459 PMC2361933

